# Supplementary material for: Genes possibly related to symbiosis in early life stages of Acropora tenuis inoculated with Symbiodinium microadriaticum
Source: Commun Biol. 2023 Oct 18;6:1027. doi: 10.1038/s42003-023-05350-8 (PMC10584924; doi:10.1038/s42003-023-05350-8)
Supplement: Supplementary file 2 — Supplementary Information [file 42003_2023_5350_MOESM2_ESM.pdf]

Supplementary Information for

Genes possibly related to symbiosis in early life stages of *Acropora tenuis* inoculated with *Symbiodinium microadriaticum*

Yuki Yoshioka [1, 2*], Yi-Ling Chiu [1], Taiga Uchida [1], Hiroshi Yamashita [3], Go Suzuki [3], and Chuya Shinzato [1*]

[1] Atmosphere and Ocean Research Institute, The University of Tokyo, Kashiwa, Chiba, Japan

[2] Marine Genomics Unit, Okinawa Institute of Science and Technology Graduate University, Onna, Okinawa, Japan

[3] Fisheries Technology Institute, Japan Fisheries Research and Education Agency, Ishigaki, Okinawa, Japan

[*] Corresponding author: Yuki Yoshioka (y.yoshioka@oist.jp) and Chuya Shinzato (c.shinzato@aori.u-tokyo.ac.jp)

Supplementary Tables

Supplementary Tables 1 pp. 2

Supplementary Tables 2 pp. 3

Supplementary Figures

Supplementary Fig. 1 pp. 4

Supplementary Fig. 2 pp. 5

Supplementary Fig. 3 pp. 6

Supplementary Fig. 4 pp. 7

Supplementary Fig. 5 pp. 8

Supplementary Fig. 6 pp. 9

Supplementary Fig. 7 pp. 10

Supplementary Fig. 8 pp. 11

Supplementary Fig. 9 pp. 12

Supplementary Fig. 10 pp. 13

Supplementary Fig. 11 pp. 14

Supplementary Fig. 12 pp. 15

Supplementary Fig. 13 pp. 16

Supplementary Fig. 14 pp. 17

Supplementary Fig. 15 pp. 18

References pp. 19

Supplementary Table 1. Numbers of Smic acquired by *Acropora tenuis*.

Developmental stages	Uptake efficiency (Average ± SD)	No. of algae in corals (Average ± SE)	Sampling time[1]	Reference
Planula larvae	93.3 ± 5.8%	11.7 ± 2.4 cells/larva	4 dpi	Yamashita et al. 2018
	93.3 ± 11.5%	55.4 ± 6.8 cells/larva	8 dpi	
	90 ± 10%	185.1 ± 23.3 cells/larva	12 dpi	
Primary polyps	100%	1138.9 ± 249 cells/polyp	10 dpi	This study
	100%	4241.7 ± 872 cells/polyp	20 dpi	

[1] dpi indicates days post-inoculation.

Supplementary Table 2. Summary of numbers of clean reads, mapped reads to *Acropora tenuis* gene models, and expressed genes.

DRA accession	Samples	No. of read after QC	Total base of read after QC	Mapping rate
DRR411688	10dpi cont1	29,408,584	1,465,416,109	74.0%
DRR411689	10dpi cont2	13,283,612	659,495,489	59.8%
DRR411690	10dpi cont3	8,107,499	403,425,697	61.4%
DRR411691	10dpi smic1	6,090,892	302,942,762	56.7%
DRR411692	10dpi smic2	7,325,435	363,895,387	55.0%
DRR411693	10dpi smic3	9,223,493	459,279,240	63.2%
DRR411694	20dpi cont1	9,402,985	467,715,310	64.5%
DRR411695	20dpi cont2	8,390,335	417,559,557	63.0%
DRR411696	20dpi smic1	8,646,344	430,486,994	58.6%
DRR411697	20dpi smic2	6,786,510	337,603,727	60.1%
	Average	10,666,569	530,782,027	61.6%

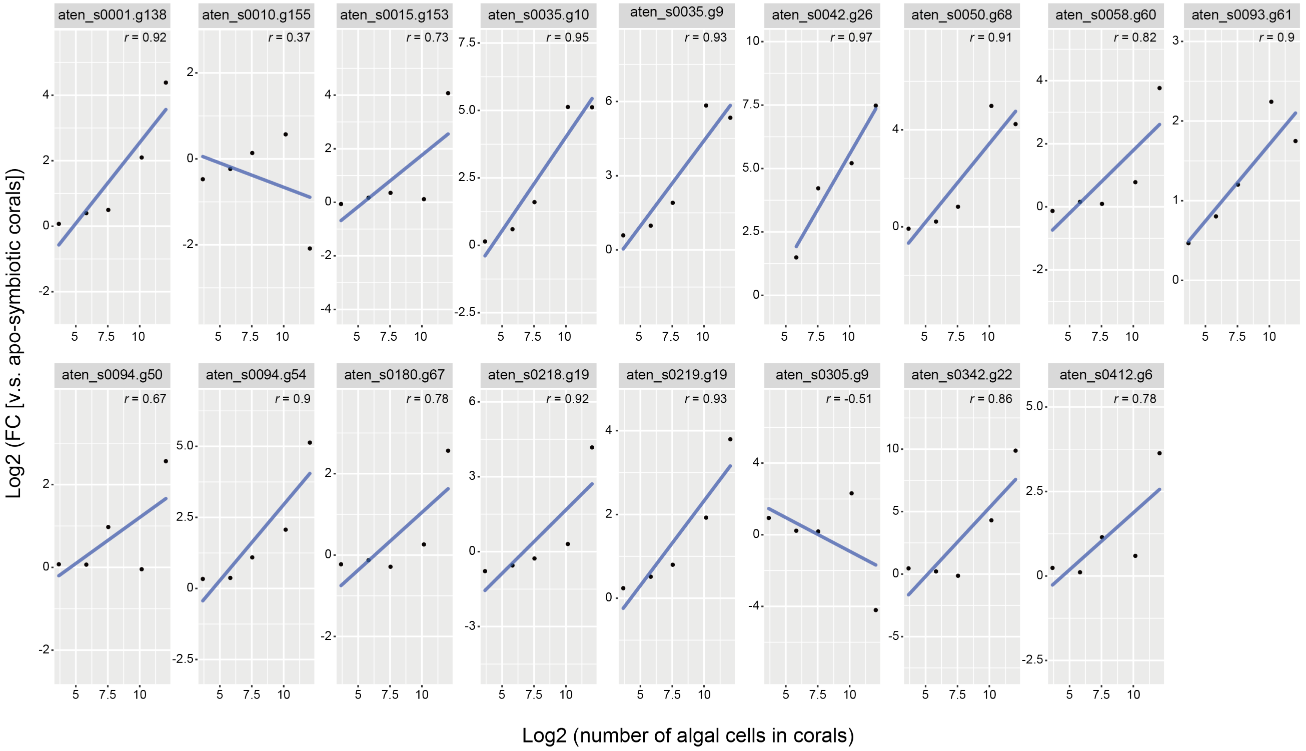

Supplementary Fig. 1. Correlation analysis between gene expression levels and algal symbiont number in corals.

Correlation between $\log_2$ (relative gene expression levels of corals inoculated with Smic) and $\log_2$ (number of algal cells in corals) were examined using the function "cor.test" in R. Gene IDs are shown at the top of each box. *r* indicates Pearson correlation. Blue line indicates regression line drawn with the function "stat_smoothe (method = lm)" in ggplot2 in R. Relative gene expression levels of planula larvae and primary polyps are provided in Supplementary Data 1. Numbers of algal cell numbers in corals are provided in Supplementary Table 1.

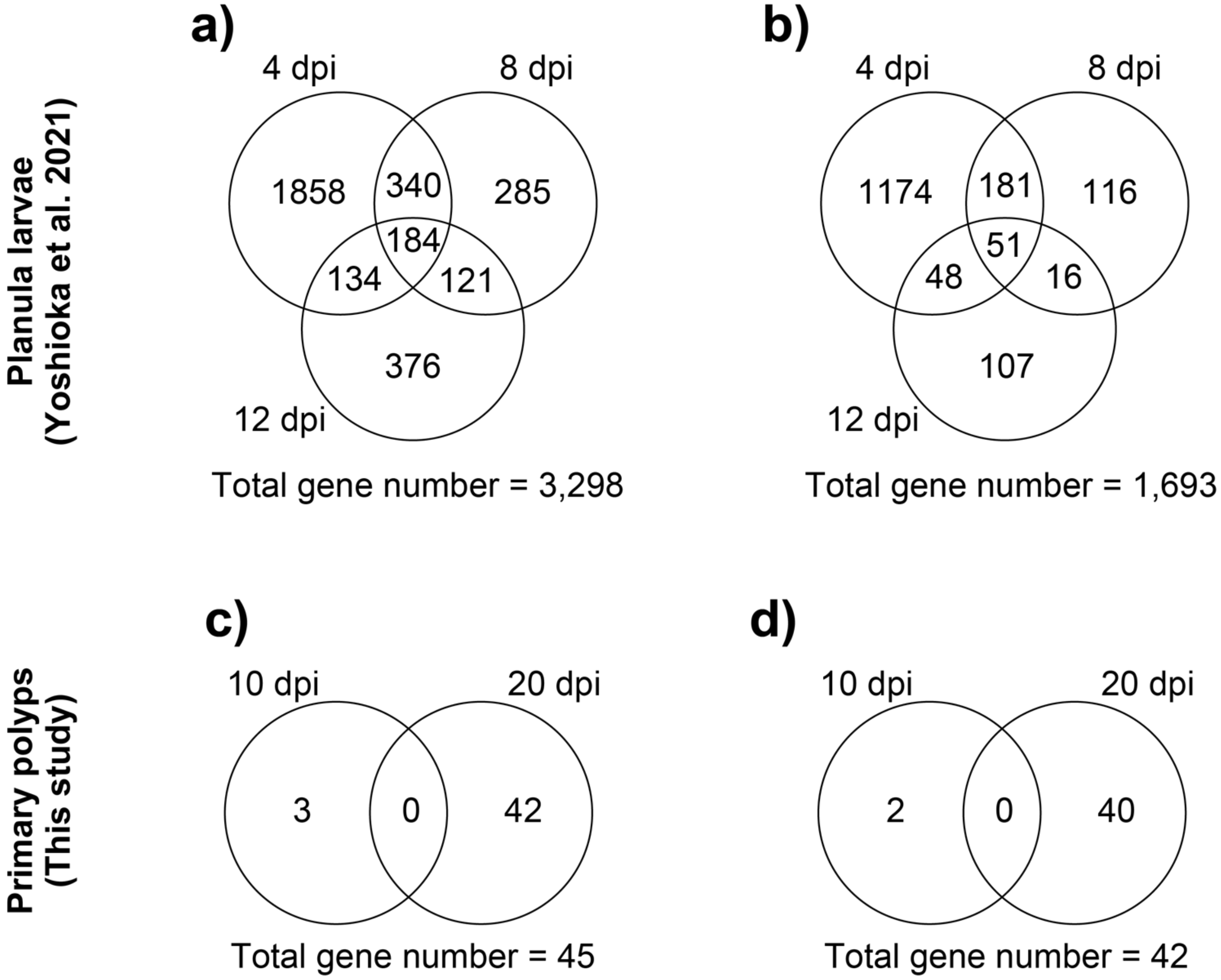

Supplementary Fig. 2. Summary of genes for which expression levels differed by age (days).

a) Venn-diagram of the number of DEGs restricted to planula larvae inoculated with Smic.

b) Venn-diagram of the number of DEGs restricted to planula larvae inoculated with Smic after removal of genes for which expression depends on age of planula larvae between 4, 8, and 12 dpi.

c) Venn-diagram of the number of DEGs restricted to primary polyps inoculated with Smic.

d) Venn-diagram of the number of DEGs restricted to primary polyps inoculated with Smic after removal of genes for which expression differs by age of primary polyps between 10 and 20 dpi.

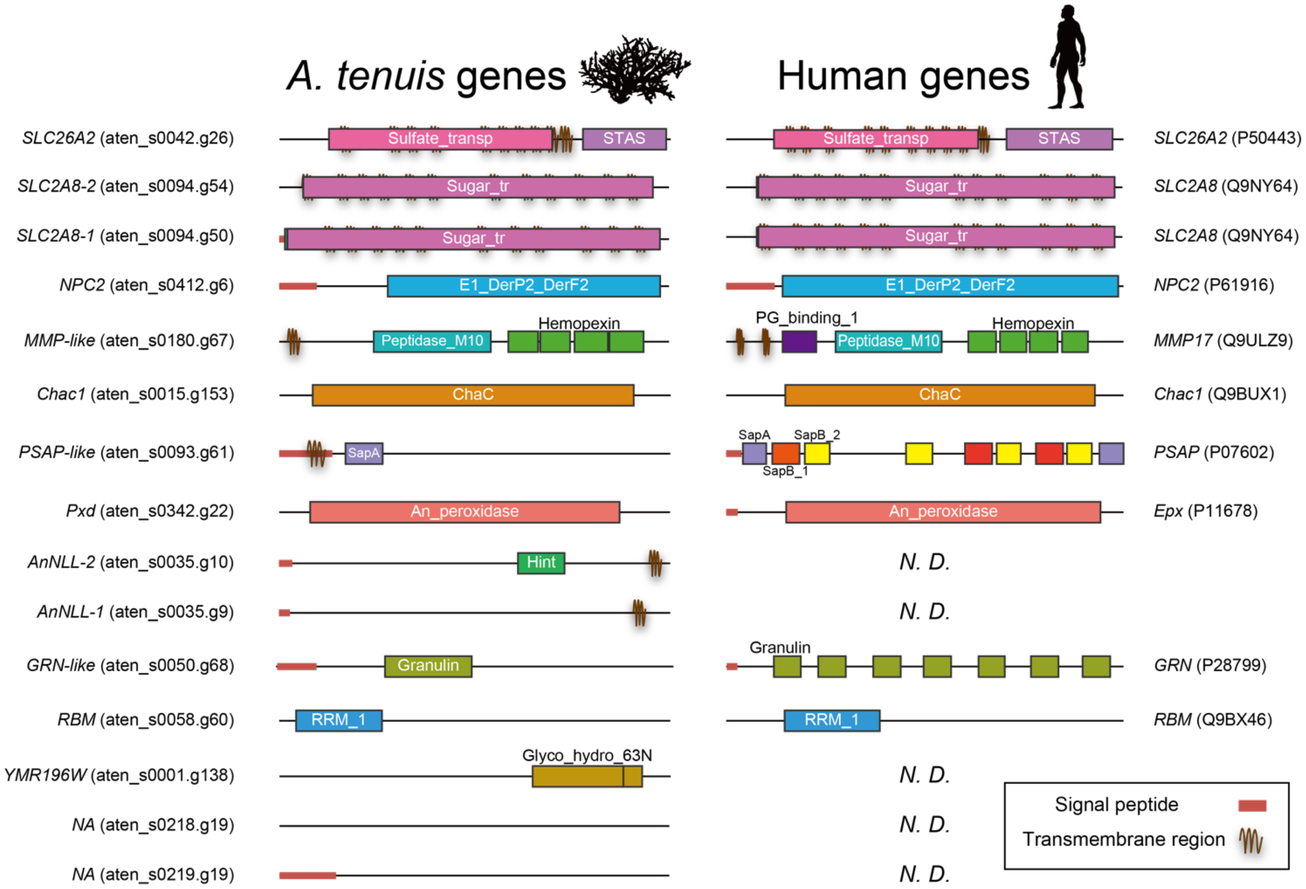

Supplementary Fig. 3. Comparison of domain composition of 15 core symbiosis-related genes with human genes.

Signal peptides, transmembrane regions, and evolutionally conserved protein domains are colored separately. Short names of protein domains are shown around protein domains. Gene names and UniProt IDs for *A. tenuis* and human are provided at left and right, respectively. Species icons are from phylopic.org.

Uncharacterized protein (*YMR196W*)
(Orthogroup 1830)

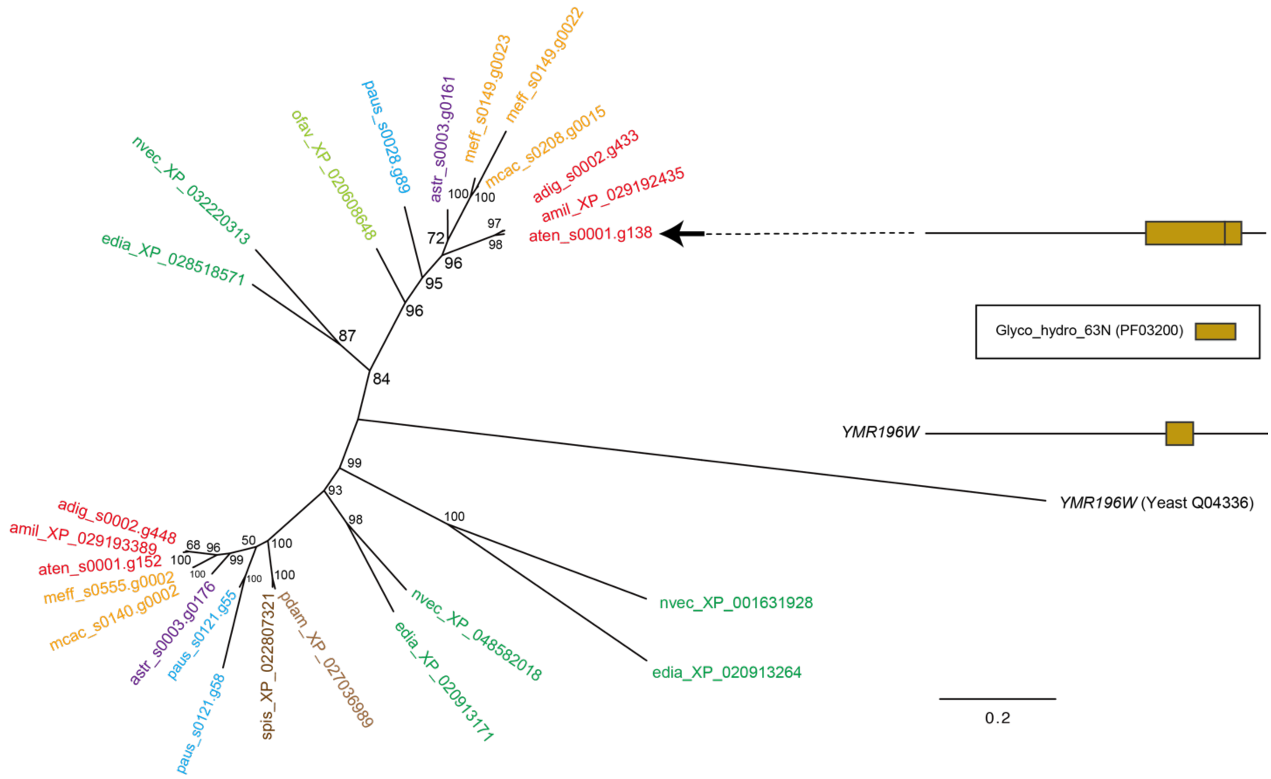

Supplementary Fig. 4. Molecular phylogenetic analysis of an uncharacterized protein (*YMR196W*).

897 gap-trimmed, aligned, amino acids were used for phylogenetic tree construction (LG model). Genes are color coded: *Acropora* (red), *Montipora* (yellow), *Astreopora* (purple), *Porites* (light-blue), *Stylophora* (brown), *Pocillopora* (light-brown), *Orbicella* (light-green), sea anemones (green), and yeast (black). Species names are abbreviated: *A. tenuis* (aten), *A. digitifera* (adig), *A. millepora* (amil), *M. cactus* (mcac), *M. efflorescens* (meff), *Astreopora myriophthalma* (astr), *Porites australiensis* (paus), *S. pistillata* (spis), *O. faveolata* (ofav), *Pocillopora damicornis* (pdam), *Exaiptasia diaphana* (edia), and *Nematostella vectensis* (nvec). Gene IDs are shown following species names. A black arrow indicates the DEG. Sequence features of the DEG and the reference gene (Yeast *YMR196W*) is shown. Note that these sequence features do not reflect sequence lengths.

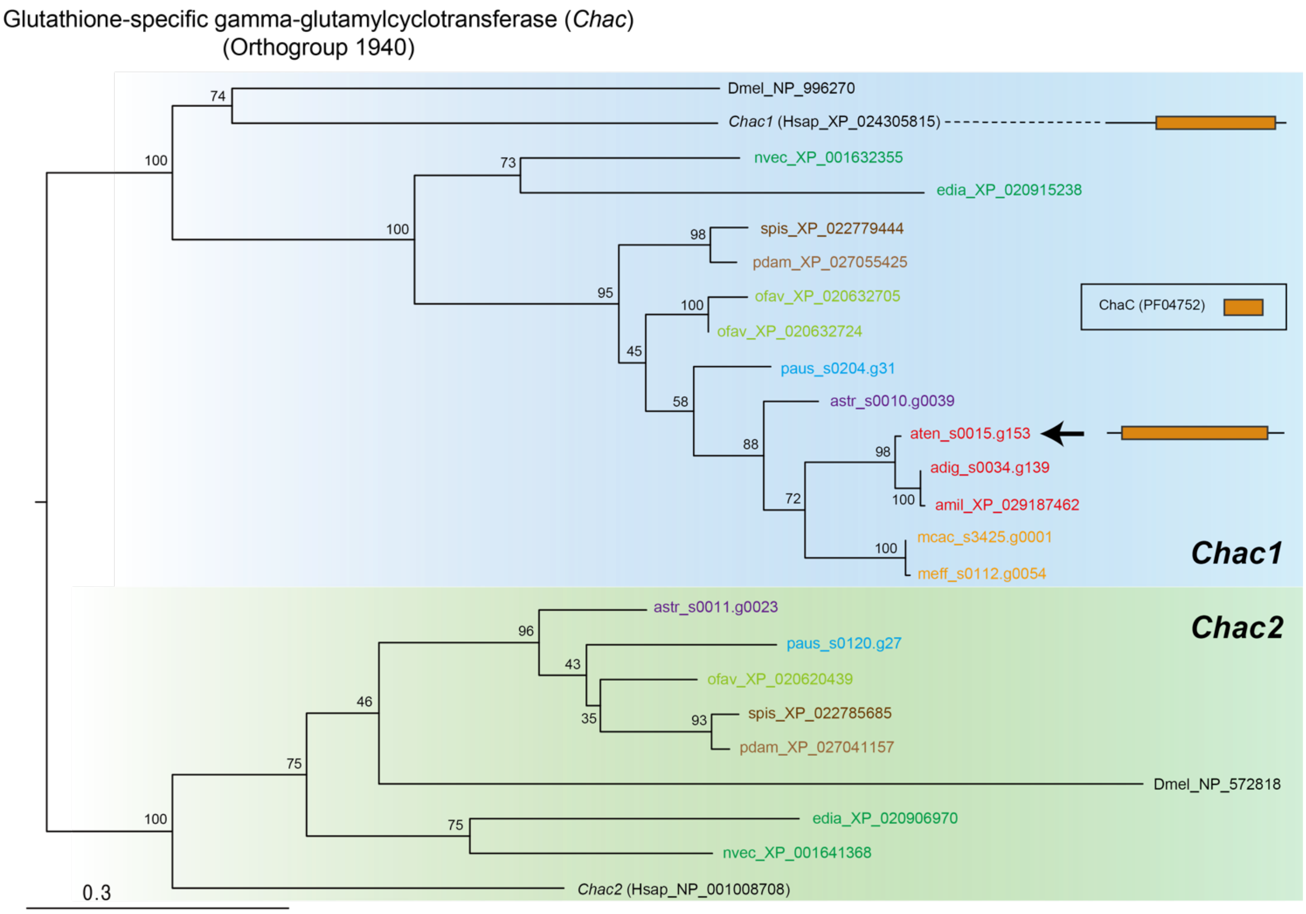

Supplementary Fig. 5. Molecular phylogenetic analysis of a glutathione-specific gamma-glutamyl cyclotransferase (*Chac*).

179 gap-trimmed, aligned amino acids were used for phylogenetic tree construction (LG model). Genes are color coded: *Acropora* (red), *Montipora* (yellow), *Astreopora* (purple), *Porites* (light-blue), *Stylophora* (brown), *Pocillopora* (light-brown), *Orbicella* (light-green), sea anemones (green), and bilaterians (black). Species names are abbreviated: *A. tenuis* (aten), *A. digitifera* (adig), *A. millepora* (amil), *M. cactus* (mcac), *M. efflorescens* (meff), *Astreopora myriophthalma* (astr), *Porites australiensis* (paus), *S. pistillata* (spis), *O. faveolata* (ofav), *Pocillopora damicornis* (pdam), *Exaiptasia diaphana* (edia), *Nematostella vectensis* (nvec), *D. melanogaster* (dmel), and *H. sapiens* (hsap). Gene IDs follow species names. A black arrow indicates the DEG. The sequence feature of the DEG and the reference gene (Human *Chac1*) are shown. Note that these sequence features do not reflect sequence lengths.

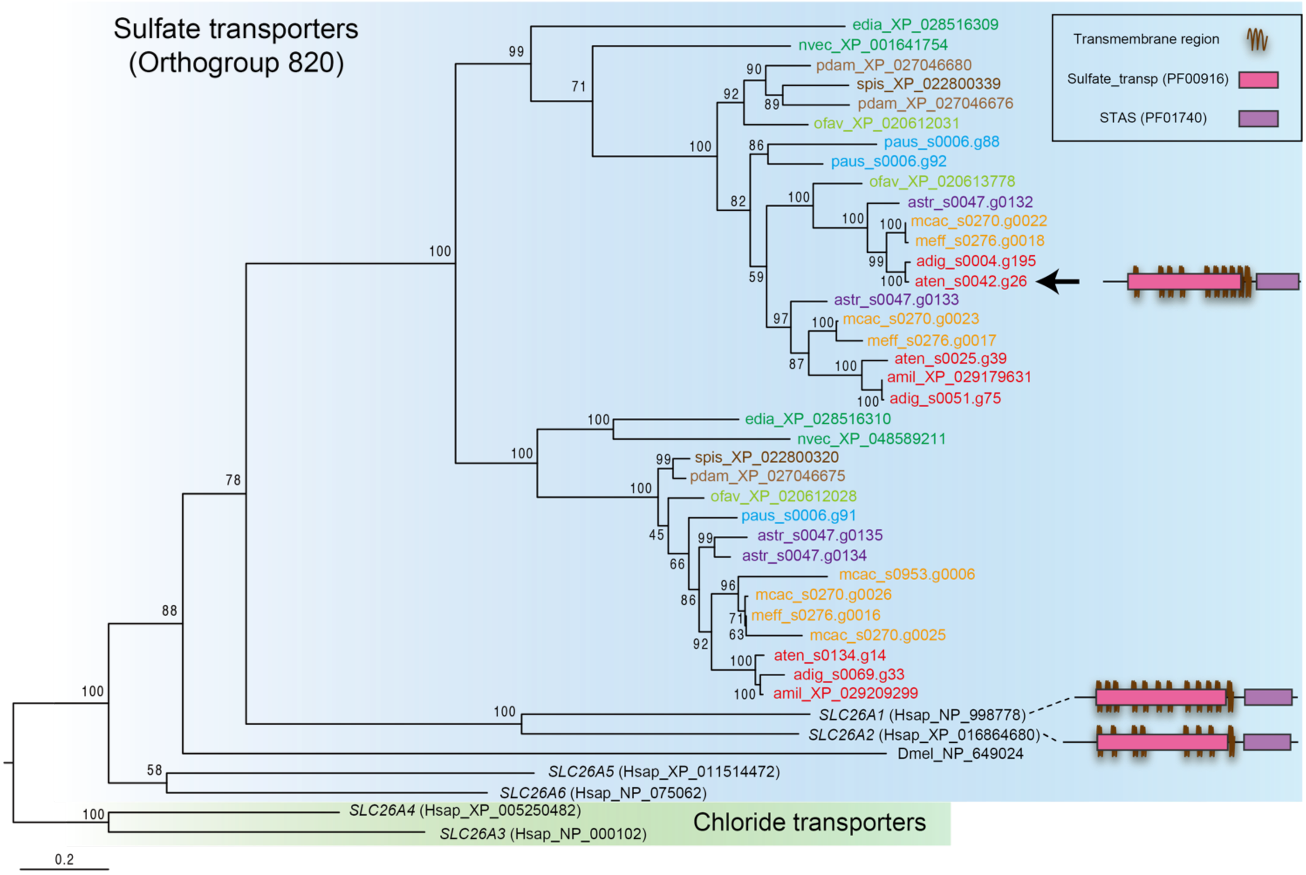

Supplementary Fig. 6. Molecular phylogenetic analysis of sulfate transporters.

603 gap-trimmed, aligned amino acids were used for phylogenetic tree construction (LG model). Genes are color coded: *Acropora* (red), *Montipora* (yellow), *Astreopora* (purple), *Porites* (light-blue), *Stylophora* (brown), *Pocillopora* (light-brown), *Orbicella* (light-green), sea anemones (green), and bilaterians (black). Species names are abbreviated: *A. tenuis* (aten), *A. digitifera* (adig), *A. millepora* (amil), *M. cactus* (mcac), *M. efflorescens* (meff), *Astreopora myriophthalma* (astr), *Porites australiensis* (paus), *S. pistillata* (spis), *O. faveolata* (ofav), *Pocillopora damicornis* (pdam), *Exaiptasia diaphana* (edia), *Nematostella vectensis* (nvec), *D. melanogaster* (dmel), and *H. sapiens* (hsap). Gene IDs follow species names. A black arrow indicates the DEG. The sequence feature of the DEG and the reference genes (Human *SLC26A1* and *SLC26A2*) is shown. The chloride transporters of *H. sapiens* were used as an outgroup. Note that these sequence features do not reflect sequence lengths.

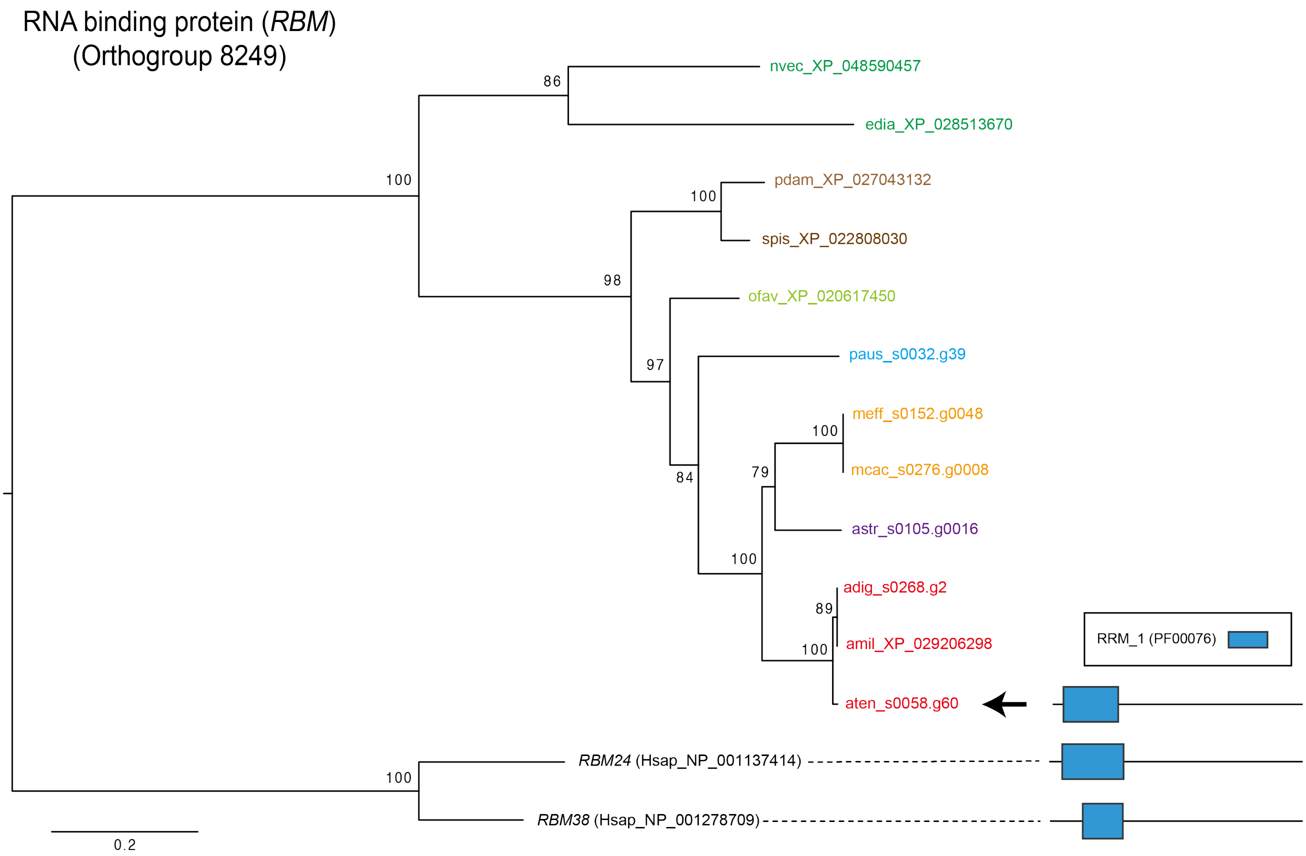

Supplementary Fig. 7. Molecular phylogenetic analysis of an RNA binding protein (*RBM*).

237 gap-trimmed, aligned amino acids were used for phylogenetic tree construction (JTT model). Genes are color coded: *Acropora* (red), *Montipora* (yellow), *Astreopora* (purple), *Porites* (light-blue), *Stylophora* (brown), *Pocillopora* (light-brown), *Orbicella* (light-green), sea anemones (green), and human (black). Species names are abbreviated: *A. tenuis* (aten), *A. digitifera* (adig), *A. millepora* (amil), *M. cactus* (mcac), *M. efflorescens* (meff), *Astreopora myriophthalma* (astr), *Porites australiensis* (paus), *S. pistillata* (spis), *O. faveolata* (ofav), *Pocillopora damicornis* (pdam), *Exaiptasia diaphana* (edia), *Nematostella vectensis* (nvec), and *H. sapiens* (hsap). Gene IDs follow species names. A black arrow indicates the DEG. The sequence feature of the DEG and the reference genes (Human *RBM24* and *RBM38*) are shown. Note that these sequence features do not reflect sequence lengths.

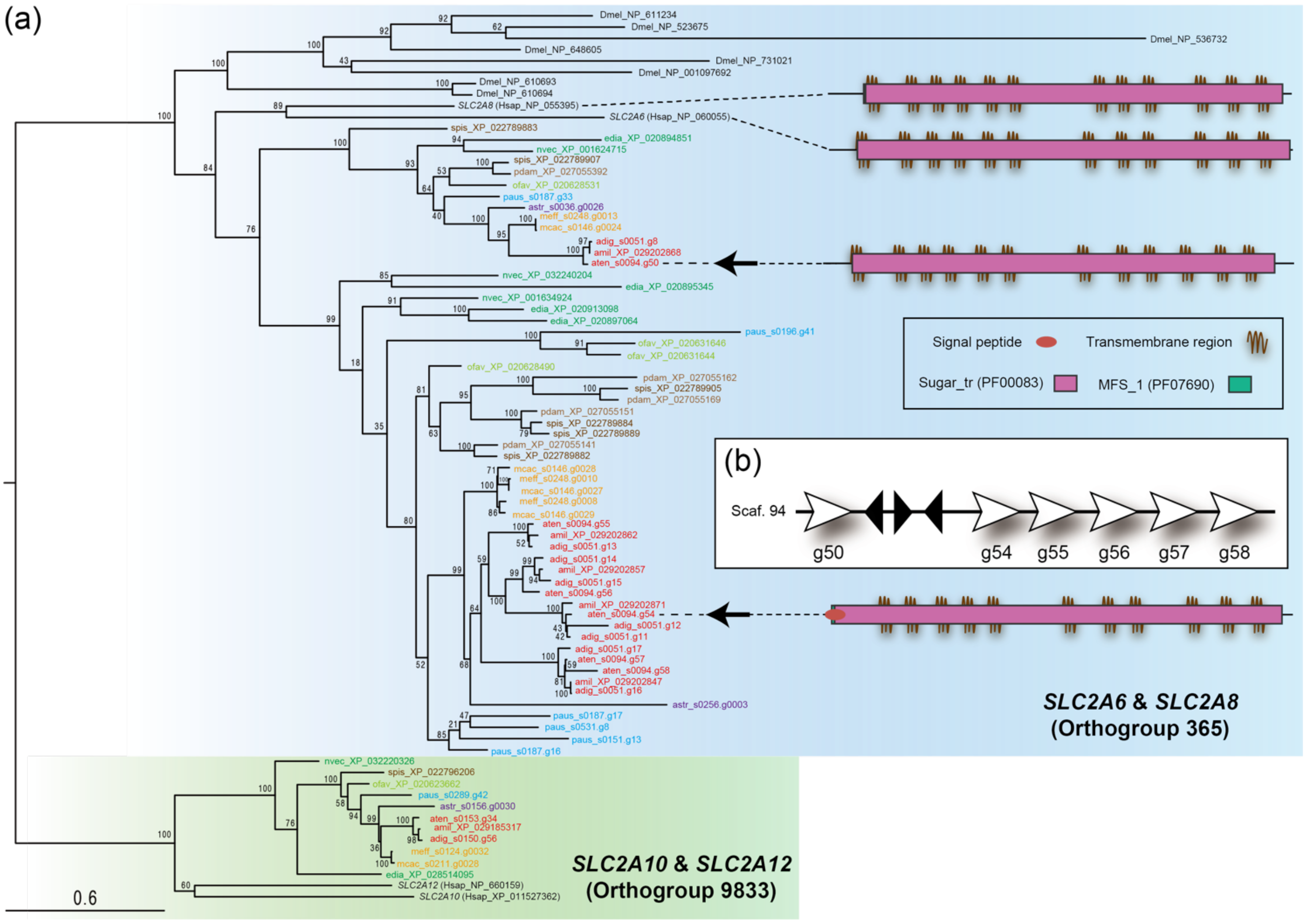

Supplementary Fig. 8. Molecular phylogenetic analysis of sugar transporters.

(a) Phylogenetic tree of sugar transporters.

468 gap-trimmed, aligned amino acids were used for phylogenetic tree construction (LG model). Genes are color coded: *Acropora* (red), *Montipora* (yellow), *Astreopora* (purple), *Porites* (light-blue), *Stylophora* (brown), *Pocillopora* (light-brown), *Orbicella* (light-green), sea anemones (green), and bilaterians (black). Species names are abbreviated: *A. tenuis* (aten), *A. digitifera* (adig), *A. millepora* (amil), *M. cactus* (mcac), *M. efflorescens* (meff), *Astreopora myriophthalma* (astr), *Porites australiensis* (paus), *S. pistillata* (spis), *O. faveolata* (ofav), *Pocillopora damicornis* (pdam), *Exaiptasia diaphana* (edia), *Nematostella vectensis* (nvec), *D. melanogaster* (dmel), and *H. sapiens* (hsap). Gene IDs follow species names. Black arrows indicate the DEGs. The sequence feature of the DEGs and the reference gene (Human *SLC2A6* and *SLC2A8*) is shown. Another sugar transporter genes (Orthogroup 9833) were used as an outgroup. Note that these sequence features do not reflect sequence lengths.

(b) Tandem duplication of sugar transporter genes in the *A. tenuis* genome.

White arrows indicate tandemly duplicated sugar transporter genes on scaffold 94 and their transcription direction.

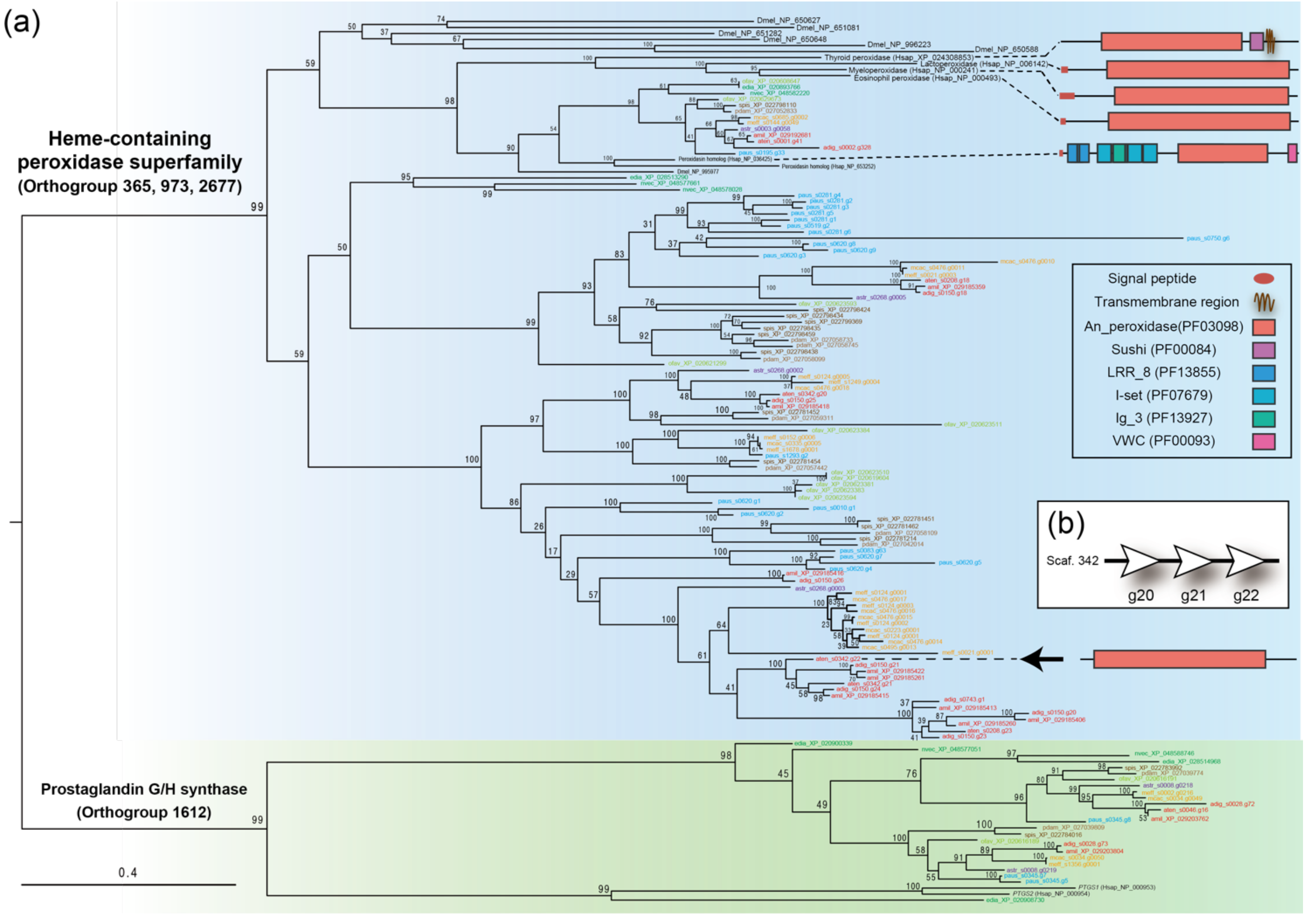

Supplementary Fig. 9. Molecular phylogenetic analysis of heme-containing peroxidase (*Pxd*) superfamily.

(a) Phylogenetic tree of *Pxd* superfamily.

503 gap-trimmed, aligned amino acids were used for phylogenetic tree construction (WAG model). Genes are color coded: *Acropora* (red), *Montipora* (yellow), *Astreopora* (purple), *Porites* (light-blue), *Stylophora* (brown), *Pocillopora* (light-brown), *Orbicella* (light-green), sea anemones (green), and bilaterians (black). Species names are abbreviated: *A. tenuis* (aten), *A. digitifera* (adig), *A. millepora* (amil), *M. cactus* (mcac), *M. efflorescens* (meff), *Astreopora myriophthalma* (astr), *Porites australiensis* (paus), *S. pistillata* (spis), *O. faveolata* (ofav), *Pocillopora damicornis* (pdam), *Exaiptasia diaphana* (edia), *Nematostella vectensis* (nvec), *D. melanogaster* (dmel), and *H. sapiens* (hsap). Gene IDs follow species names. A black arrow indicates the DEG. The sequence feature of the DEG and the reference genes (Human *TPO*, *LPO*, *MPO*, *EPO*, and *Peroxidasin-homologue*) is shown. Prostaglandin G/H synthases (Orthogroup 1612) were used as an outgroup. Note that these sequence features do not reflect sequence lengths.

(b) Tandem duplication of *Pxd* genes in *A. tenuis* genome.

White arrows indicate tandemly duplicated *Pxd* genes on scaffold 342 and their transcription direction, respectively.

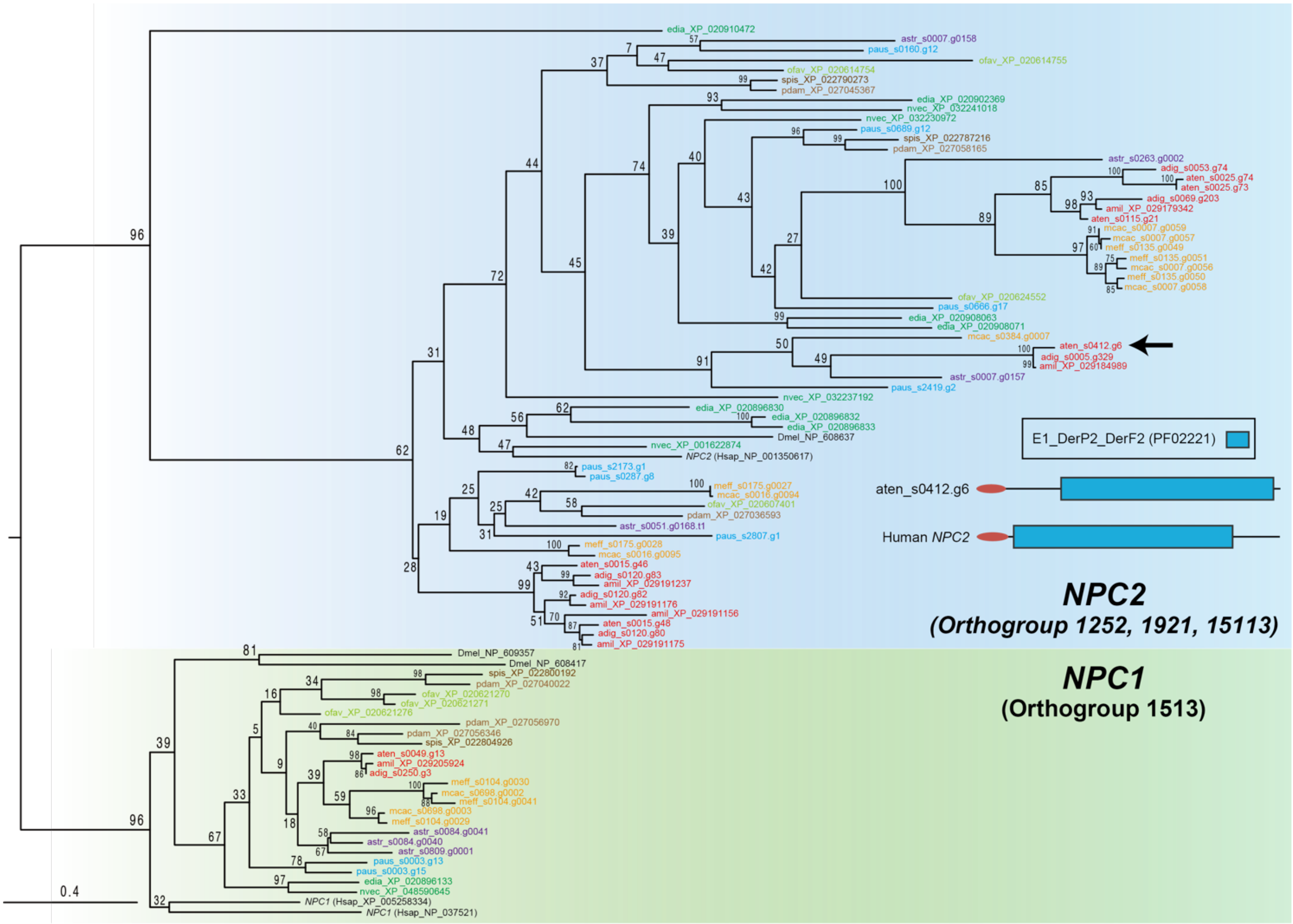

Supplementary Fig. 10. Molecular phylogenetic analysis of Niemann-pick type C2 sterol transporters (*NPC2*) 148 gap-trimmed, aligned amino acids were used for phylogenetic tree construction (WAG model). Genes are color coded: *Acropora* (red), *Montipora* (yellow), *Astreopora* (purple), *Porites* (light-blue), *Stylophora* (brown), *Pocillopora* (light-brown), *Orbicella* (light-green), sea anemones (green), and human (black). Species names are abbreviated: *A. tenuis* (aten), *A. digitifera* (adig), *A. millepora* (amil), *M. cactus* (mcac), *M. efflorescens* (meff), *Astreopora myriophthalma* (astr), *Porites australiensis* (paus), *S. pistillata* (spis), *O. faveolata* (ofav), *Pocillopora damicornis* (pdam), *Exaiptasia diaphana* (edia), *Nematostella vectensis* (nvec), and *H. sapiens* (hsap). Gene IDs follow species names. A black arrow indicates the DEG. The sequence feature of the DEG and the reference gene (Human *NPC2*) is shown. *NPC1* genes (Orthogroup 1513) were used as an outgroup. Note that these sequence features do not reflect sequence lengths.

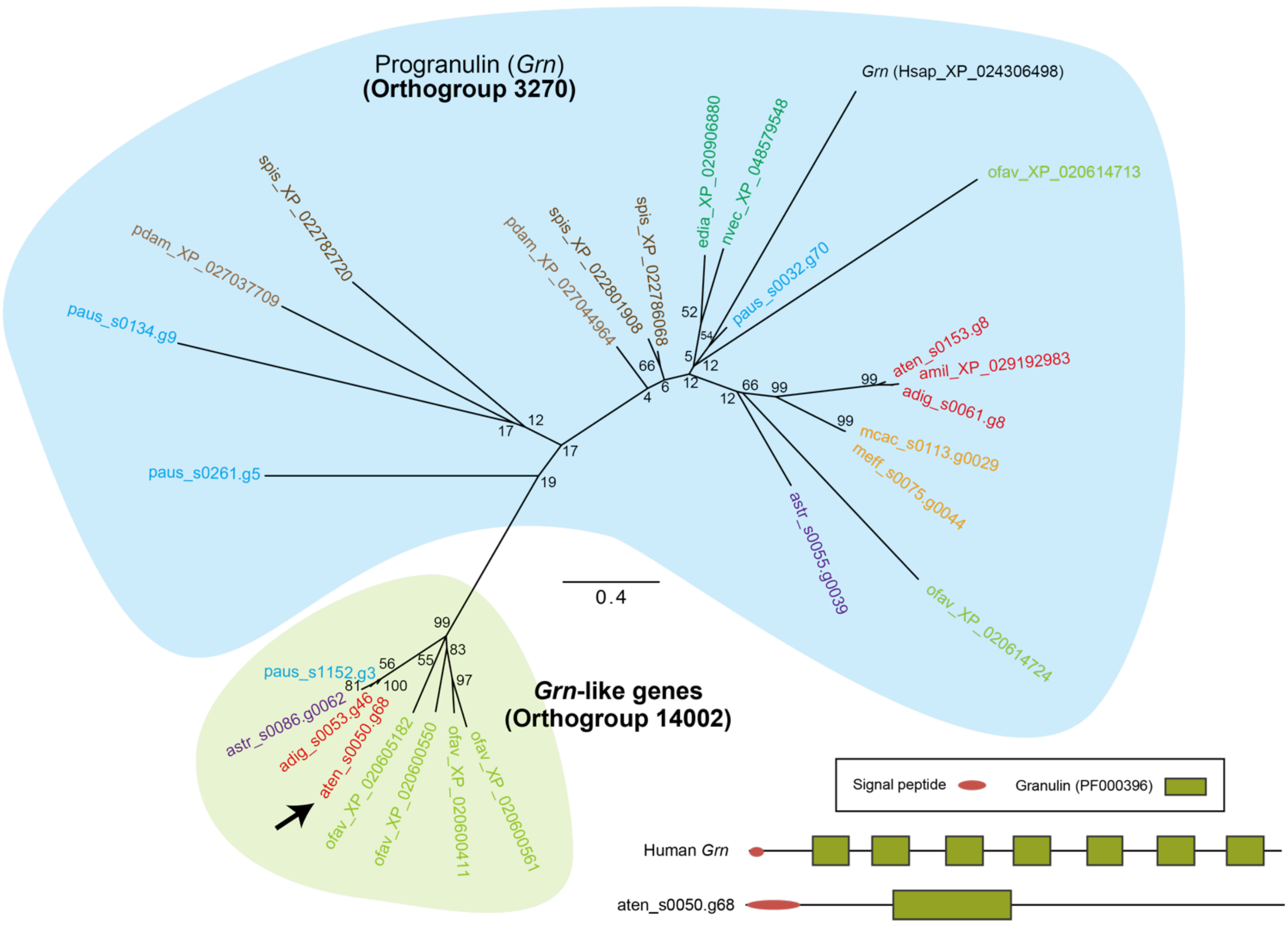

Supplementary Fig. 11. Molecular phylogenetic analysis of progranulin-like genes (*Grn*).

177 gap-trimmed, aligned amino acids were used for phylogenetic tree construction (FLU model). Genes are color coded: *Acropora* (red), *Montipora* (yellow), *Astreopora* (purple), *Porites* (light-blue), *Stylophora* (brown), *Pocillopora* (light-brown), *Orbicella* (light-green), sea anemones (green), and human (black). Species names are abbreviated: *A. tenuis* (aten), *A. digitifera* (adig), *A. millepora* (amil), *M. cactus* (mcac), *M. efflorescens* (meff), *Astreopora myriophthalma* (astr), *Porites australiensis* (paus), *S. pistillata* (spis), *O. faveolata* (ofav), *Pocillopora damicornis* (pdam), *Exaiptasia diaphana* (edia), *Nematostella vectensis* (nvec), and *H. sapiens* (hsap). Gene IDs follow species names. A black arrow indicates the DEG. The sequence feature of the DEG and the reference gene (Human *Grn*) is shown. Note that these sequence features do not reflect sequence lengths.

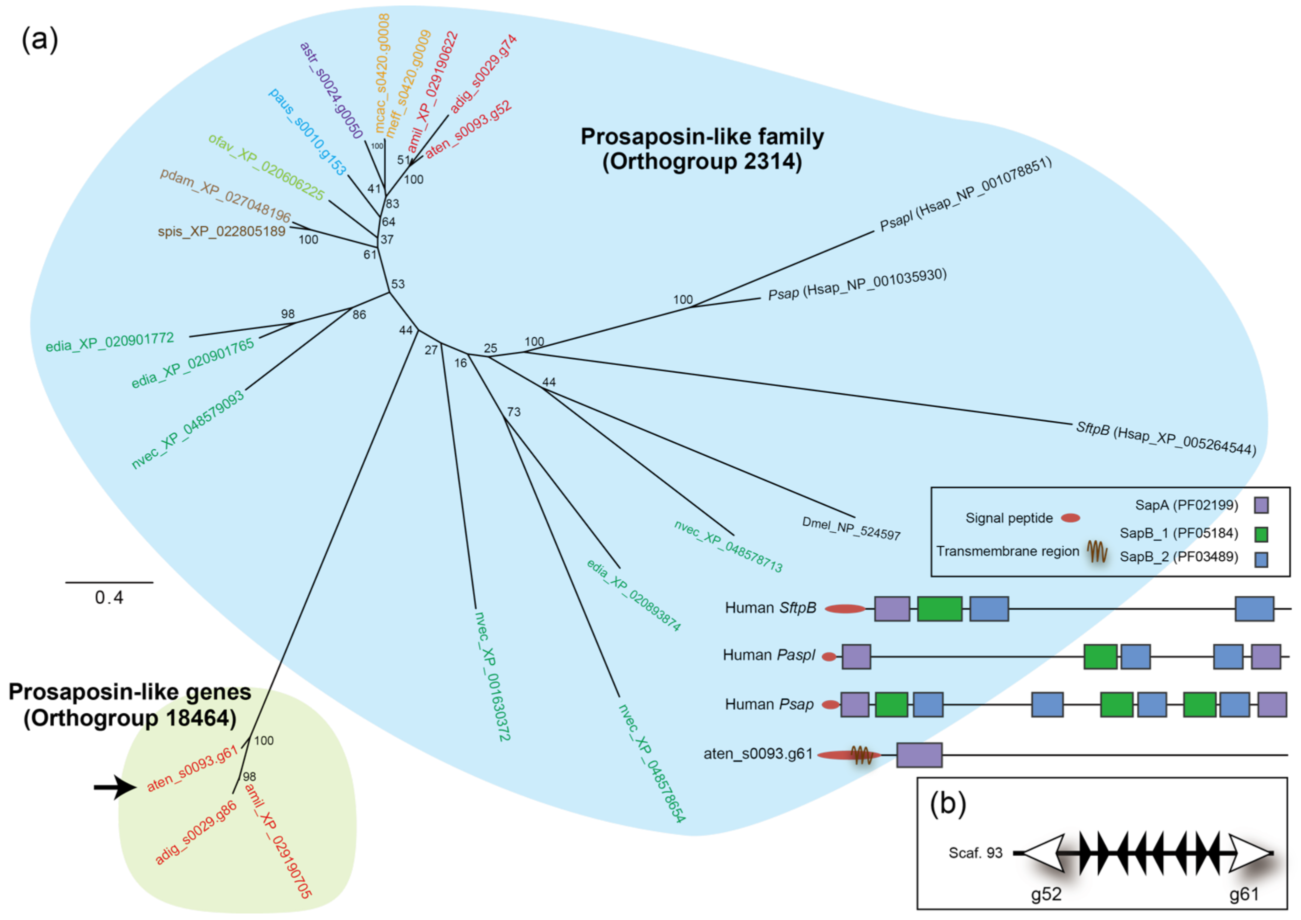

Supplementary Fig. 12. Molecular phylogenetic analysis of the prosaposin-like family.

(a) Phylogenetic tree of prosaposin-like genes.

385 gap-trimmed, aligned amino acids were used for phylogenetic tree construction (FLU model). Genes are color coded: *Acropora* (red), *Montipora* (yellow), *Astreopora* (purple), *Porites* (light-blue), *Stylophora* (brown), *Pocillopora* (light-brown), *Orbicella* (light-green), sea anemones (green), and bilaterians (black). Species names are abbreviated: *A. tenuis* (aten), *A. digitifera* (adig), *A. millepora* (amil), *M. cactus* (mcac), *M. efflorescens* (meff), *Astreopora myriophthalma* (astr), *Porites australiensis* (paus), *S. pistillata* (spis), *O. faveolata* (ofav), *Pocillopora damicornis* (pdam), *Exaiptasia diaphana* (edia), *Nematostella vectensis* (nvec), *D. melanogaster* (dmel), and *H. sapiens* (hsap). Gene IDs follow species names. A black arrow indicates the DEG. Sequence features of the DEG and the reference genes (Human *SftpB*, *Paspl*, and *Psap*) is shown. Note that these sequence features do not reflect sequence lengths.

(b) Tandem duplication of *Psap*-like genes in *A. tenuis* genome.

White arrows indicate tandemly duplicated *Psap*-like genes on scaffold 93 and their transcription direction.

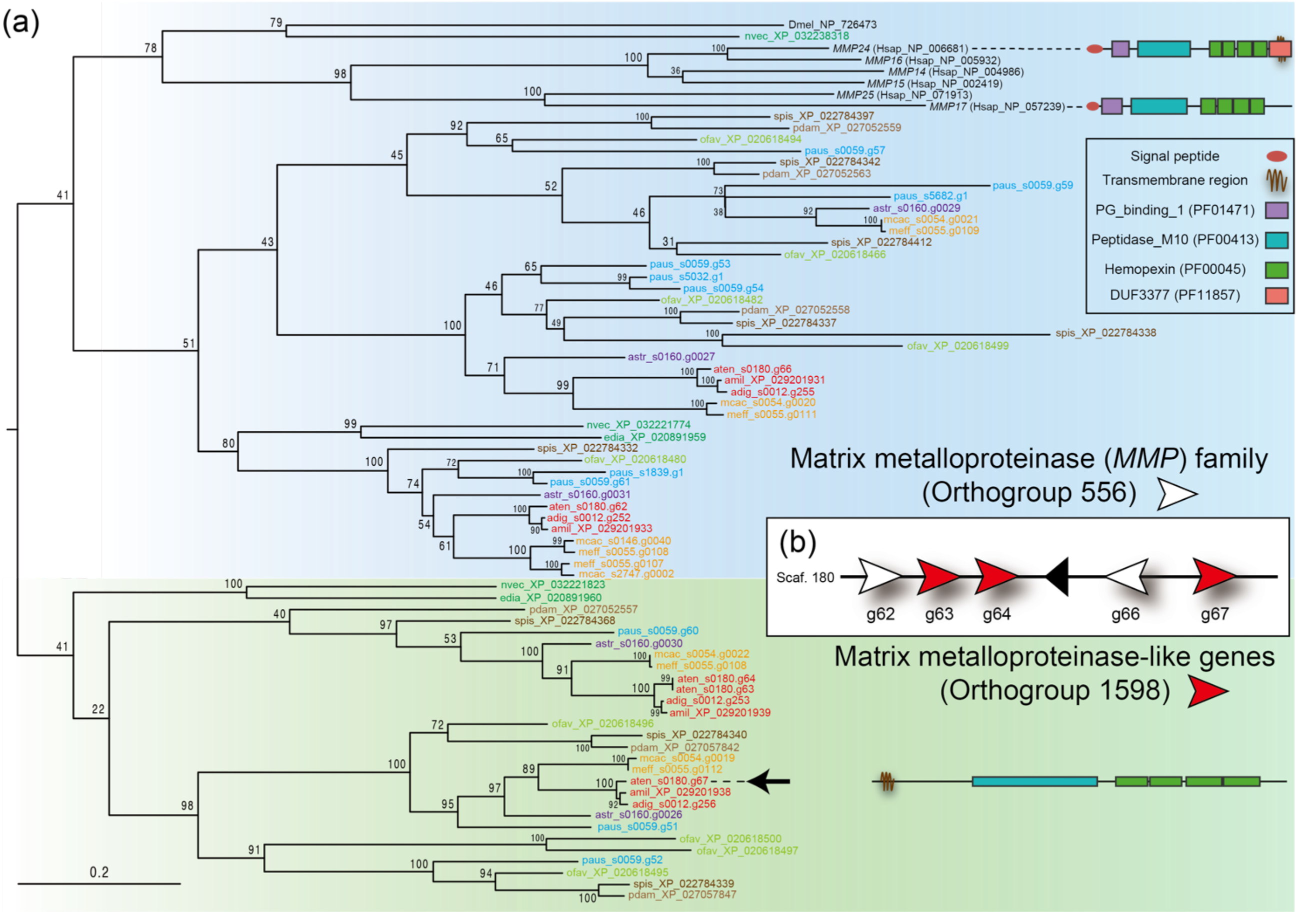

Supplementary Fig. 13. Molecular phylogenetic analysis of the matrix metalloproteinase (*MMP*) family.

(a) Phylogenetic tree of the *MMP* family.

409 gap-trimmed, aligned amino acids were used for phylogenetic tree construction (WAG model). Genes are color coded: *Acropora* (red), *Montipora* (yellow), *Astreopora* (purple), *Porites* (light-blue), *Stylophora* (brown), *Pocillopora* (light-brown), *Orbicella* (light-green), sea anemones (green), and bilaterians (black). Species names are abbreviated: *A. tenuis* (aten), *A. digitifera* (adig), *A. millepora* (amil), *M. cactus* (mcac), *M. efflorescens* (meff), *Astreopora myriophthalma* (astr), *Porites australiensis* (paus), *S. pistillata* (spis), *O. faveolata* (ofav), *Pocillopora damicornis* (pdam), *Exaiptasia diaphana* (edia), *Nematostella vectensis* (nvec), *D. melanogaster* (dmel), and *H. sapiens* (hsap). Gene IDs follow species names. A black arrow indicates the DEG. The sequence feature of the DEG and the reference genes (Human *MMP24* and *MMP25*) is shown. Note that these sequence features do not reflect sequence lengths.

(b) Tandem duplication of the *MMP* and *MMP*-like genes in *A. tenuis* genome.

Red and white arrows indicate tandemly duplicated *MMP* and *MMP*-like genes on scaffold 180, respectively, and their transcription direction.

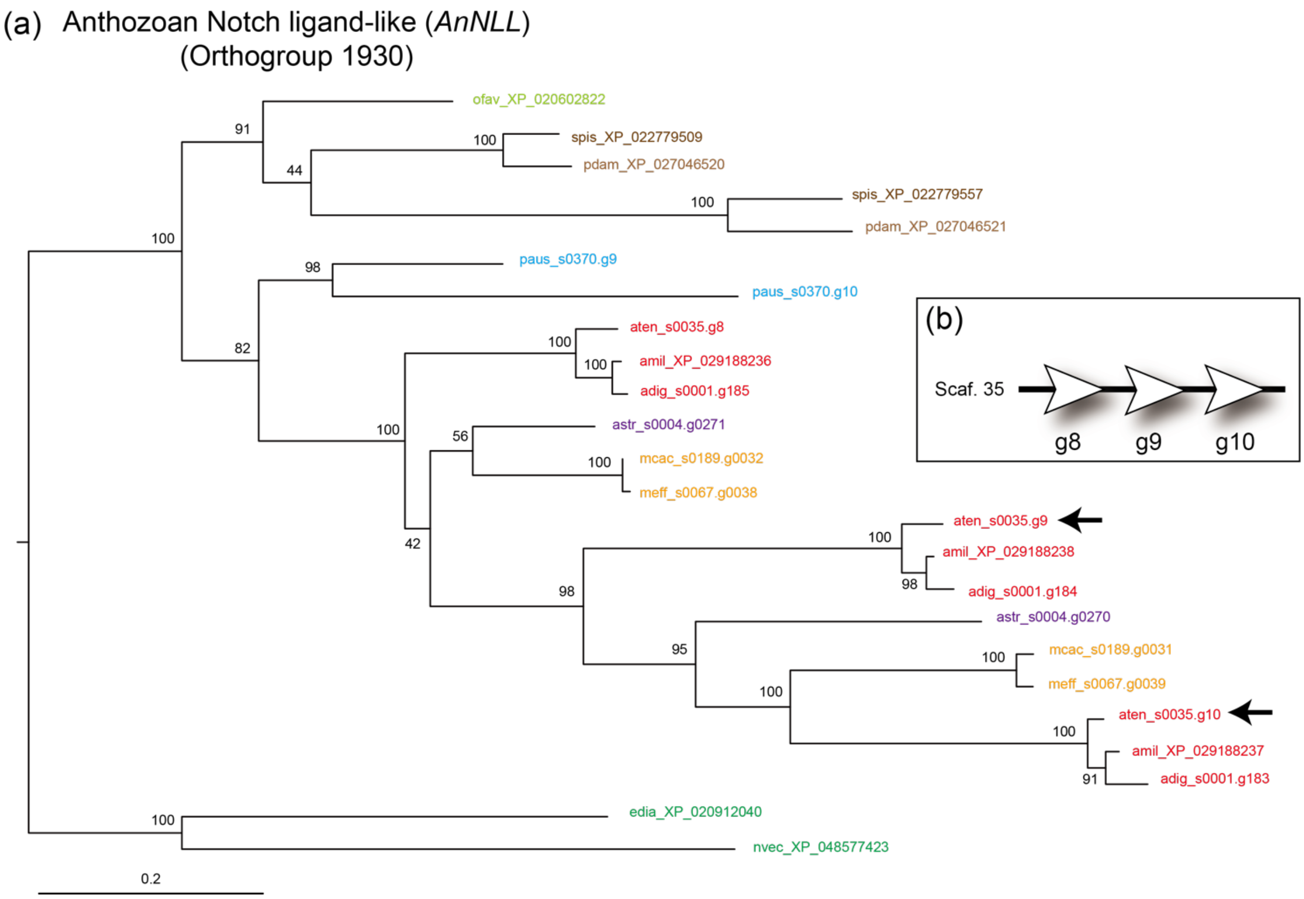

Supplementary Fig. 14. Molecular phylogenetic analysis of anthozoan Notch ligand-like (*AnNLL*).

(a) Phylogenetic tree of *AnNLL*.

573 gap-trimmed, aligned amino acids were used for phylogenetic tree construction (FLU model). Genes are color coded: *Acropora* (red), *Montipora* (yellow), *Astreopora* (purple), *Porites* (light-blue), *Stylophora* (brown), *Pocillopora* (light-brown), *Orbicella* (light-green), and sea anemones (green). Species names are abbreviated: *A. tenuis* (aten), *A. digitifera* (adig), *A. millepora* (amil), *M. cactus* (mcac), *M. efflorescens* (meff), *Astreopora myriophthalma* (astr), *Porites australiensis* (paus), *S. pistillata* (spis), *O. faveolata* (ofav), *Pocillopora damicornis* (pdam), *Exaiptasia diaphana* (edia), and *Nematostella vectensis* (nvec). Gene IDs follow species names. Black arrows indicate DEGs.

(b) Tandem duplication of *AnNLL* in *A. tenuis* genome.

White arrows indicate tandemly duplicated *AnNLL* on scaffold 35 and their transcription direction.

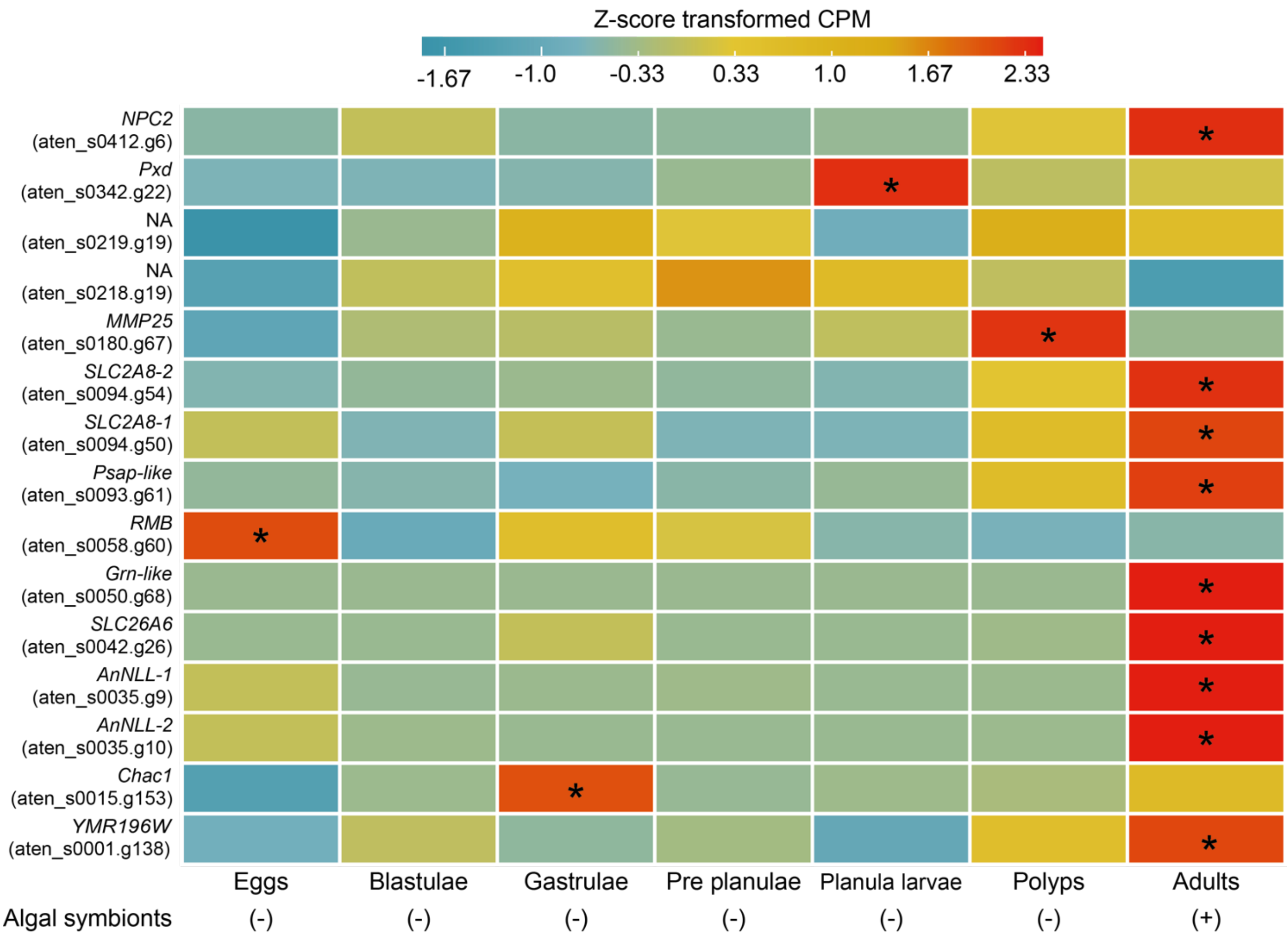

Supplementary Fig. 15. Comparative expression of the 15 genes in various samples from eggs to an adult colony in *Acropora tenuis*.

Minus and plus indicate the absence or presence of algal symbiont in samples, respectively. Asterisks indicate significantly higher expression (Z-score > 1.96).

References

Yamashita, H., Suzuki, G., Shinzato, C., Jimbo, M. & Koike, K. Symbiosis process between Acropora larvae and Symbiodinium differs even among closely related Symbiodinium types. *Mar Ecol Prog Ser* **592**, 119-128 (2018).

Yoshioka, Y. *et al*. Whole-Genome Transcriptome Analyses of Native Symbionts Reveal Host Coral Genomic Novelties for Establishing Coral-Algae Symbioses. *Genome Biol Evol* **13**, doi:10.1093/gbe/evaa240 (2021).
