# Supplementary material for: Genes possibly related to symbiosis in early life stages of Acropora tenuis inoculated with Symbiodinium microadriaticum
Source: Commun Biol. 2023 Oct 18;6:1027. doi: 10.1038/s42003-023-05350-8 (PMC10584924; doi:10.1038/s42003-023-05350-8)
Supplement: Supplementary file 3 — Description of Supplementary Materials [file 42003_2023_5350_MOESM3_ESM.docx]

**Description of Additional Supplementary Files**

**File name:** Supplementary Data 1

**Description:** The list of differentially expressed genes in primary polyps and planula larvae inoculated with Symbiodinium microadriaticum
