# Supplementary material for: Genes possibly related to symbiosis in early life stages of Acropora tenuis inoculated with Symbiodinium microadriaticum
Source: Commun Biol. 2023 Oct 18;6:1027. doi: 10.1038/s42003-023-05350-8 (PMC10584924; doi:10.1038/s42003-023-05350-8)
Supplement: Supplementary file 5 — Reporting Summary [file 42003_2023_5350_MOESM5_ESM.pdf]

nature portfolio

Corresponding author(s): Yuki Yoshioka, Chuya Shinzato

Last updated by author(s): August 31, 2023

Reporting Summary

Nature Portfolio wishes to improve the reproducibility of the work that we publish. This form provides structure for consistency and transparency in reporting. For further information on Nature Portfolio policies, see our Editorial Policies and the Editorial Policy Checklist.

Statistics

For all statistical analyses, confirm that the following items are present in the figure legend, table legend, main text, or Methods section.

n/a	Confirmed	
☐	☒	The exact sample size (n) for each experimental group/condition, given as a discrete number and unit of measurement
☐	☒	A statement on whether measurements were taken from distinct samples or whether the same sample was measured repeatedly
☐	☒	The statistical test(s) used AND whether they are one- or two-sided<br>*Only common tests should be described solely by name; describe more complex techniques in the Methods section.*
☒	☐	A description of all covariates tested
☐	☒	A description of any assumptions or corrections, such as tests of normality and adjustment for multiple comparisons
☒	☐	A full description of the statistical parameters including central tendency (e.g. means) or other basic estimates (e.g. regression coefficient) AND variation (e.g. standard deviation) or associated estimates of uncertainty (e.g. confidence intervals)
☒	☐	For null hypothesis testing, the test statistic (e.g. F, t, r) with confidence intervals, effect sizes, degrees of freedom and P value noted<br>*Give P values as exact values whenever suitable.*
☒	☐	For Bayesian analysis, information on the choice of priors and Markov chain Monte Carlo settings
☒	☐	For hierarchical and complex designs, identification of the appropriate level for tests and full reporting of outcomes
☐	☒	Estimates of effect sizes (e.g. Cohen's d, Pearson's r), indicating how they were calculated

Our web collection on statistics for biologists contains articles on many of the points above.

Software and code

Policy information about availability of computer code

Data collection	N/A
Data analysis	CUTADAPT v1.16; BWA v0.7.17; SALMON v1.5.2; EdgeR v3.32.1; R v4.0.3; Blast v2.2.28; InterProScan v5.27-66.0; TransDecoder v5.5.0; Orthofinder v2.5.4; MAFFT v7.310; trimAL v1.2; RAxML v8.2.10

For manuscripts utilizing custom algorithms or software that are central to the research but not yet described in published literature, software must be made available to editors and reviewers. We strongly encourage code deposition in a community repository (e.g. GitHub). See the Nature Portfolio guidelines for submitting code & software for further information.

Data

Policy information about availability of data

All manuscripts must include a data availability statement. This statement should provide the following information, where applicable:

- Accession codes, unique identifiers, or web links for publicly available datasets
- A description of any restrictions on data availability
- For clinical datasets or third party data, please ensure that the statement adheres to our policy

Raw RNA sequencing data for A. tenuis primary polyps inoculated with S. microadriaticum and with no exposure of algal cells are deposited in the DDBJ/EMBL/GenBank databases under accession number DRA014957 (BioProject accession: PRJDB8332).

April 2023

Research involving human participants, their data, or biological material

Policy information about studies with human participants or human data. See also policy information about sex, gender (identity/presentation), and sexual orientation and race, ethnicity and racism.

Reporting on sex and gender	N/A
Reporting on race, ethnicity, or other socially relevant groupings	N/A
Population characteristics	N/A
Recruitment	N/A
Ethics oversight	N/A

Note that full information on the approval of the study protocol must also be provided in the manuscript.

Field-specific reporting

Please select the one below that is the best fit for your research. If you are not sure, read the appropriate sections before making your selection.

☒ Life sciences ☐ Behavioural & social sciences ☐ Ecological, evolutionary & environmental sciences

For a reference copy of the document with all sections, see nature.com/documents/nr-reporting-summary-flat.pdf

Life sciences study design

All studies must disclose on these points even when the disclosure is negative.

Sample size	For transcriptomic analysis, we obtained an average of ten million RNA-seq reads, which is sufficient to detect differentially expressed genes, as discussed in Liu et al. 2014 (doi: https://doi.org/10.1093/bioinformatics/btt688).
Data exclusions	N/A
Replication	We performed algal inoculation experiments using Acropora tenuis primary polyps, with at least two replicates per treatment.
Randomization	N/A
Blinding	N/A

Reporting for specific materials, systems and methods

We require information from authors about some types of materials, experimental systems and methods used in many studies. Here, indicate whether each material, system or method listed is relevant to your study. If you are not sure if a list item applies to your research, read the appropriate section before selecting a response.

Materials & experimental systems

n/a	Involved in the study	
☒	☐	Antibodies
☒	☐	Eukaryotic cell lines
☒	☐	Palaeontology and archaeology
☐	☒	Animals and other organisms
☒	☐	Clinical data
☒	☐	Dual use research of concern
☒	☐	Plants

Methods

n/a	Involved in the study	
☒	☐	ChIP-seq
☒	☐	Flow cytometry
☒	☐	MRI-based neuroimaging

Animals and other research organisms

Policy information about studies involving animals; ARRIVE guidelines recommended for reporting animal research, and Sex and Gender in Research

Laboratory animals	N/A
Wild animals	We used the coral Acropora tenuis. After the experiment, adult coral colonies were returned to the original sea (Sekisei Lagoon, Okinawa, Japan) in 2022.
Reporting on sex	N/A
Field-collected samples	All coral polyps were maintained at a concentration of ~5 individuals/mL of artificial sea water (ASW) prepared with Viesalt (Marine Tech) at 25°C.
Ethics oversight	The care and use of experimental animals are compiled with Japanese animal welfare laws, guidelines, and policies as approved by the university guidelines. Permits for coral collection were kindly provided by the Okinawa Prefectural Government (Permits 3-67 [1]).

Note that full information on the approval of the study protocol must also be provided in the manuscript.
